# Safety, Efficacy, and Immunogenicity of a Multivalent Adjuvanted *Staphylococcus aureus* Vaccine in Adults With Recent Skin and Soft Tissue Infections: An Observer-Blind, Randomized, Placebo-Controlled, Multinational Phase 1/2 Trial

**DOI:** 10.1093/cid/ciag162

**Published:** 2026-03-09

**Authors:** Eugene Athan, Richard N Greenberg, David A Baker, Ruchir Shah, Siddharth Dubhashi, Aysha Badat, Claire Thurlow, Joel Schlessinger, Adam Wronski, Marcin Zakrzewski, David Turner, Ilaria Bindi, Venere Basile, Bruno Galletti, Fabiana Spensieri, Michela Brazzoli, Giulia Zigon, Giulia Luna Cilio, Giulia Ranzato, Maria Lattanzi, Michele Pellegrini, for the Staph aureus 208833 study group, Katherine Garnham, Fiona Bisshop, Mark Bloch, Olga Voloshyna, Brajesh Gupta, Kirankumar Jadhav, Krina Patel, Penelope Montgomery, Renate Koops, Joanne Finlay, Michal Adamczyk, Shaunagh Emanuel, Rosie Mngqibisa, Loren Miller, Scott Striplin, David Dougherty, Nirav Shah

**Affiliations:** Public Health Unit, Barwon Health, Deakin University, Geelong, Australia; University of Kentucky, Lexington, Kentucky, USA; East Sydney Doctors, Darlinghurst, NSW, Australia; Sanjivani Super Specialty Hospital, Ahmedabad, India; All India Institute of Medical Science, Nagpur, India; Wits Clinical Research, Soweto, Johannesburg, South Africa; Tasman, Southern Clinical Trials, Nelson, New Zealand; Advanced Skin Research Center, Omaha, Nebraska, USA; Specjalistyczny Osrodek Deramatologi, Bialystok, Poland; Nzoz Holsamed, Oddzial Libero, Katowice, Poland; Cripps Health Centre, University of Nottingham, Nottingham, United Kingdom; GSK, Siena, Italy; GSK, Siena, Italy; GSK, Siena, Italy; GSK, Siena, Italy; GSK, Siena, Italy; GSK, Siena, Italy; GSK, Siena, Italy; GSK, Siena, Italy; GSK, Siena, Italy; GSK, Siena, Italy

**Keywords:** *Staphylococcus aureus*, vaccine, efficacy, skin and soft tissue infections

## Abstract

**Background:**

A vaccine that prevents *Staphylococcus aureus* skin and soft tissue infections (SA-SSTIs) would have a major impact on public health.

**Methods:**

A 2-part randomized study began with a phase 1, first-in-human, dose escalation that tested the safety of the 5-antigen *S. aureus* vaccine (SA5Ag), half or full antigen doses, unadjuvanted or with AS01_E_ adjuvant, in 32 healthy volunteers aged 18–50 years. In the phase 2, proof-of-principle part, 194 participants aged 18–64 years with recent SA-SSTI were administered 2 full doses of AS01_E_-adjuvanted SA5Ag (SA5Ag-Adj) or placebo, 2 months apart, and followed for 12 months. Vaccine safety (primary objective), vaccine efficacy (VE; secondary/tertiary objectives), and immunogenicity (tertiary objectives) were evaluated.

**Results:**

Following a positive safety evaluation in phase 1, participants were enrolled into phase 2 until predefined futility criteria were met at the interim efficacy analysis. Twelve months post-dose 2, SA5Ag-Adj showed no efficacy in preventing recurrent SA-SSTIs (VE, −38.1%; 95% confidence interval, −245.8 to 40.9), despite inducing robust functional immune responses against 3 (*S. aureus* capsular polysaccharide types 5 and 8 [CP5 and CP8], alpha-hemolysin [Hla]) of the 5 vaccine antigens. Solicited local adverse events (AEs) were more frequent in the SA5Ag-Adj group versus the placebo group but were mostly mild or moderate in intensity. Frequencies of medically attended AEs and serious AEs were similar across groups.

**Conclusions:**

In participants with recent history of SA-SSTI, SA5Ag-Adj vaccine had an acceptable safety profile and induced robust functional immune responses against CP5, CP8, and Hla antigens but did not reduce the rate of recurrent SA-SSTIs at 12 months from last dose.

**Clinical Trial Registration.** NCT04420221.

## Teaser

The AS01_E_-adjuvanted 5-antigen *Staphylococcus aureus* vaccine had an acceptable safety profile in healthy participants and those with recent history of *S. aureus* skin and soft tissue infections (SA-SSTIs) but did not induce protection against recurrent episodes of SA-SSTI.

*Staphylococcus aureus* is a major bacterial pathogen and the leading cause of skin and soft tissue infections (SSTIs) [1, 2]. United States (US) claims data showed an SSTI incidence rate of 77.5 per 1000 person-years, with recurrences for 26% of index cases, despite strategies such as decolonization, hygiene awareness, and antimicrobial prophylaxis protocols [3]. *Staphylococcus aureus* SSTIs (SA-SSTIs) are a major contributor to antibiotic consumption worldwide [4, 5], and *S*. *aureus* is a leading cause of deaths associated with antimicrobial resistance [6, 7].

A vaccine that prevents SA-SSTIs would fulfill a critical unmet medical need. Several vaccine candidates elicited protective immune responses against different *S*. *aureus* antigens in preclinical studies but did not prove efficacious in selected target patient populations [8–14]. The V710 *S. aureus* vaccine, which contains nonadjuvanted iron surface determinant B, failed to demonstrate efficacy against staphylococcal infections in cardiothoracic surgery patients [9]. Phase 3 studies of a vaccine that contains *S. aureus* capsular polysaccharide types 5 and 8 (CP5, CP8) in end-stage renal disease patients showed a trend for short-term (up to 40 weeks) efficacy against *S. aureus* bacteremia [10], not confirmed by primary endpoint analysis following 1 or 2 vaccine doses [10, 11]. A vaccine that contains a recombinant adhesin protein that is structurally similar to *S. aureus* clumping factor A (ClfA) tested in military recruits failed to prevent nasal/oral acquisition [14]. Finally, *S. aureus* infections were not prevented with a 4-antigen vaccine that contains CP5, CP8, ClfA, and a recombinant nonlipidated version of manganese transporter C in patients undergoing spinal surgery [12]. Multivalent vaccine formulations that also include adjuvants have therefore been explored with the intent to cover different *S*. *aureus* determinants of pathogenicity and induce cell-mediated immune (CMI) responses, potentially maximizing the chances of showing clinical efficacy [15].

Here, we describe the main results from a phase 1/2 clinical study of a pentavalent vaccine developed by GSK, SA5Ag, that targets multiple virulence mechanisms with key and complementary roles in *S*. *aureus* infections [16]. SA5Ag contains staphylococcal protein A (SpA), a major immune-evasion factor; alpha-hemolysin (Hla), a pore-forming toxin; anti-phagocytic CP5 and CP8; and ClfA, a virulence factor that mediates fibrinogen binding. Four antigens are bioconjugates (CP5-Hla, CP8-ClfA), and the lead vaccine candidate (SA5Ag-Adj), tested in phase 2, contains AS01_E_ adjuvant [17]. This study evaluated vaccine safety and reactogenicity, immunogenicity, and performed a preliminary evaluation of the clinical efficacy of 2 SA5Ag-Adj doses administered 2 months apart in adults with recent SA-SSTI history.

## METHODS

### Study Design

This phase 1/2, randomized, controlled, observer-blinded study (NCT04420221) was conducted between June 2020 and March 2024 at 42 sites in Australia, India, New Zealand, Poland, South Africa, the United Kingdom, and the United States. It was approved by independent institutional ethics committees of participating sites and conducted in accordance with the Declaration of Helsinki and International Council for Harmonization guidelines for Good Clinical Practice. Written informed consent was provided by all participants.

### Participants

The first part of the study (phase 1) included healthy participants aged 18–50 years, and the proof-of-principle study part (phase 2) enrolled participants aged 18–64 years who had an SSTI with *S*. *aureus*–positive culture, documented within 30 days before informed consent, that was treated before randomization until clinical resolution (ie, no signs/symptoms of ongoing infection, such as swelling, erythema, pain, or drainage). The main inclusion and exclusion criteria are provided in the Supplementary Material; these are listed in full in the study register (https://www.gsk-studyregister.com/trials/208833).

### Randomization, Masking, and Procedures

There were 4 phase 1 study groups, each included 8 participants with a 3:1 randomization ratio between investigational vaccine formulations and placebo (see Figure 1). Phase 2 vaccinations were performed using a staggered approach: the first 40 participants were randomly allocated (3:1) to receive 2 doses, 2 months apart, of SA5Ag-Adj or placebo. After safety evaluation, continued enrollment was planned with random allocation (1:1) to vaccine or placebo. Group allocation was conducted via a central randomization system, using a minimization procedure to account for site and previous SSTIs (yes/no) in the last 12 months.

**Figure 1.**
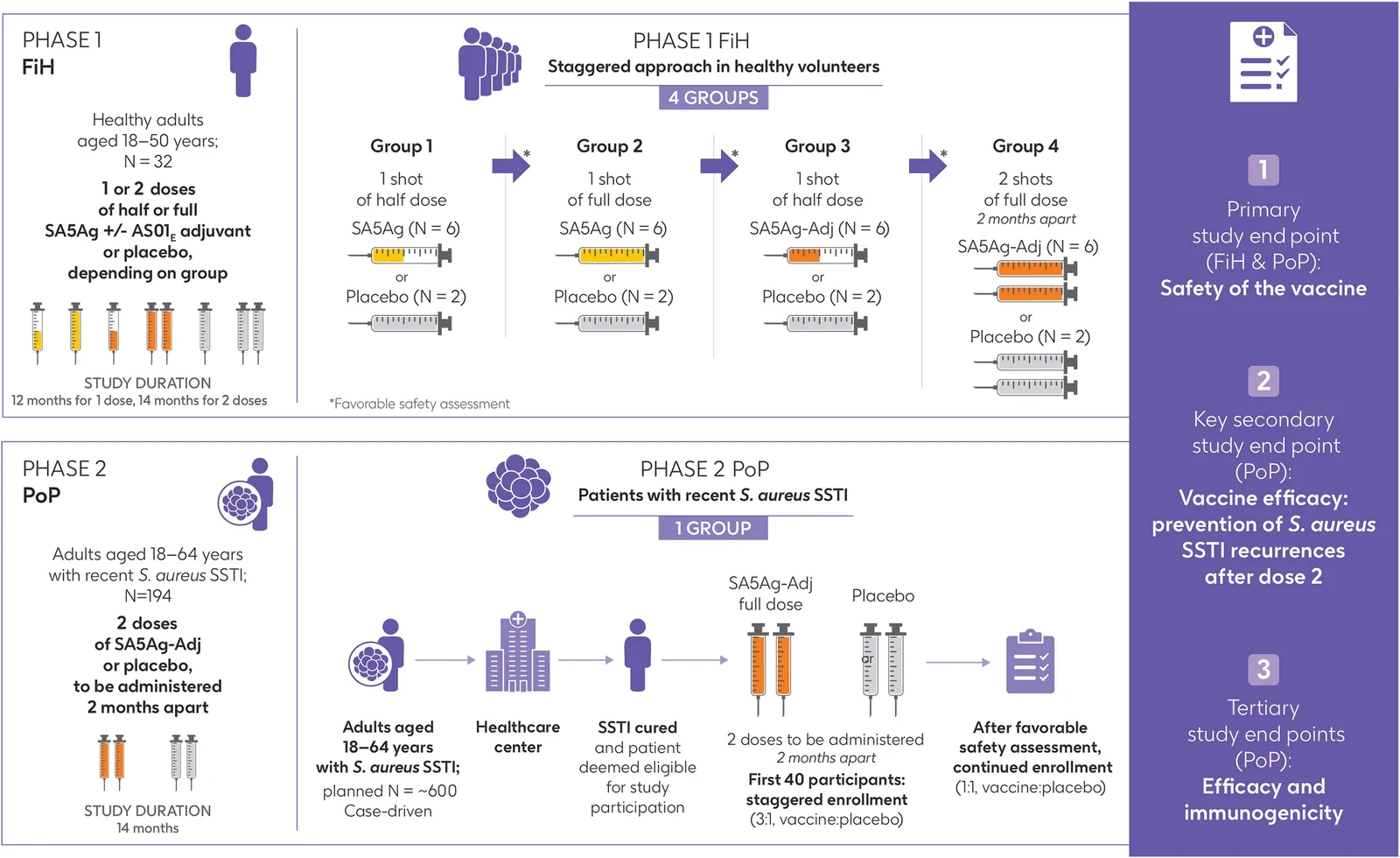
Overview of the study design. *Recent *Staphylococcus aureus* SSTI infection: culture confirmed SA-SSTI within 30 days before providing informed consent to participate in the study. Abbreviations: FiH, first-in-human; N, number of participants; PoP, proof-of-principle; SA5Ag, nonadjuvanted 5-antigen *Staphylococcus aureus* vaccine formulation; SA5Ag-Adj, SA5Ag vaccine formulation adjuvanted with AS01_E_; SSTI, skin and soft tissue infection.

The study was observer-blind, that is, participants, investigators, and outcomes assessors were blinded to group treatment. Study staff accountable for handling, preparing, and administering study doses were unblinded but were not involved in any study assessment. All data reports were prepared by an external independent data analysis center (IDAC) for evaluation by the independent safety review committee. An unblinded futility analysis was performed by the IDAC, which made recommendations on further study conduct.

The vaccine formulations administered in each study phase are specified in Figure 1, and their composition is described in the Supplementary Material. The placebo was a 0.9% saline solution. All doses were administered by intramuscular injection in the deltoid of the nondominant arm.

### Study Outcomes

The primary study objective was to assess the reactogenicity and safety of SA5Ag vaccine formulations, with endpoints including percentages of participants who reported solicited local (pain, erythema, swelling at the injection site) or systemic (headache, fatigue, nausea, vomiting, diarrhea, abdominal pain, myalgia, shivering, fever) adverse events (AEs), recorded in electronic diaries for 7 days after each dose (see the Supplementary Material). Unsolicited AEs were reported up to 30 days after each dose, and serious AEs (SAEs), medically attended AEs, and potential immune-mediated disease (pIMD) were reported in the 12 months following the last study dose.

The secondary aim was to evaluate vaccine efficacy (VE) in preventing recurrent culture-confirmed SA-SSTIs. The number of participants with at least 1 culture-confirmed SA-SSTI that occurred from 14 days after the second SA5Ag-Adj dose, up to the following 12 months, was the key secondary endpoint. The same analysis was performed for cases reported from 14 days after the first dose. Tertiary endpoints included the number of participants in these time periods with at least 1 culture-confirmed or suspected SA-SSTI, time to first SA-SSTI recurrence, and number of recurrences. Descriptive assessment of vaccine immunogenicity (summarized in the Supplementary Material) was planned as a tertiary study objective and will be reported in full elsewhere. Serum levels of interleukin-2 (IL-2) and IL-17A were calculated at baseline. CMI analyses were performed for a subset of 24 participants who consented to additional blood samples.

### Statistical Analyses

There was no formal sample size calculation for phase 1. Phase 2 had an event-driven group sequential design that enabled an interim analysis for efficacy/futility, as described below (see also the Supplementary Material).

The primary analysis included participants who received study vaccination and provided safety data, which were summarized as percentages with exact 95% confidence intervals (CIs). In phase 1, safety data for participants who received 1 or 2 placebo doses were pooled.

In phase 2, analysis of the secondary endpoint for efficacy was based on the modified full analysis set (mFAS; participants who received 2 doses and provided efficacy data). Log-rank score statistics and boundary values were used to test for early stopping in the interim analysis for efficacy/futility, which was performed when 13 SA-SSTI cases were reported in the period starting 14 days after the second SA5Ag-Adj dose (see the Supplementary Material). For the key secondary endpoint, VE was calculated following a group sequential design using the O’Brien–Fleming spending function. The between-group hazard ratio and its repeated CIs were calculated using observed statistics and boundary values, with VE derived as 100 × (1 minus hazard ratio).

A final analysis for the secondary efficacy endpoint with 27 recurrent SA-SSTI events was planned, but additional enrollment and further vaccination of enrolled participants were stopped as the futility criteria were met at the interim analysis, and the planned 27 events could not be reached by end-of-study. Therefore, a descriptive final analysis was conducted on all recurrent SA-SSTI events recorded during the study, including estimates of incidence rate, hazard ratio, and VE. Descriptive analyses of immunogenicity were performed.

The data were analyzed using SAS (Viya version 03.05).

## RESULTS

### Participants

None of the 32 participants enrolled in phase 1 withdrew. In phase 2, 194 participants with a recent SA-SSTI (simple SSTIs amenable to culture, including cellulitis, erysipelas, impetigo, folliculitis, furuncles, carbuncles, abscesses, and trauma-related infections) were randomized, and 192 received the first dose (103 SA5Ag-Adj, 89 placebo) and were included in the FAS; 114 (66 SA5Ag-Adj, 48 placebo) who received both doses were included in the mFAS. Thirty-three participants withdrew, most commonly because of consent withdrawal not due to an AE (Supplementary Figure 1).

The baseline characteristics of the participants are shown in Supplementary Table 1.

### Safety: Primary Objective

Reactogenicity and safety data review during phase 1 (Table 1, Figure 2) did not raise concerns and led to enrollment of participants with recent SA-SSTIs into the second study phase. In phase 2, solicited AEs within 7 days post-vaccination were reported by 75 (73.5%) participants in the SA5Ag-Adj group and 50 (56.8%) in the placebo group. After any dose, solicited local and systemic AEs were reported by 64 (62.7%) and 66 (64.7%) participants, respectively, in the SA5Ag-Adj group and by 12 (13.6%) and 48 (54.5%), respectively, in the placebo group. In both groups, the most frequent solicited local AE was pain at the injection site, and the most common solicited systemic AEs were fatigue and headache, which was consistent with phase 1 findings (Figure 2). Overall, reported solicited AEs tended to be more frequent after the second SA5Ag-Adj dose than after the first and were generally mild/moderate in intensity, with severe solicited AEs reported by 14 (13.7%) participants in the SA5Ag-Adj group and 4 (4.5%) participants in the placebo group.

**Figure 2.**
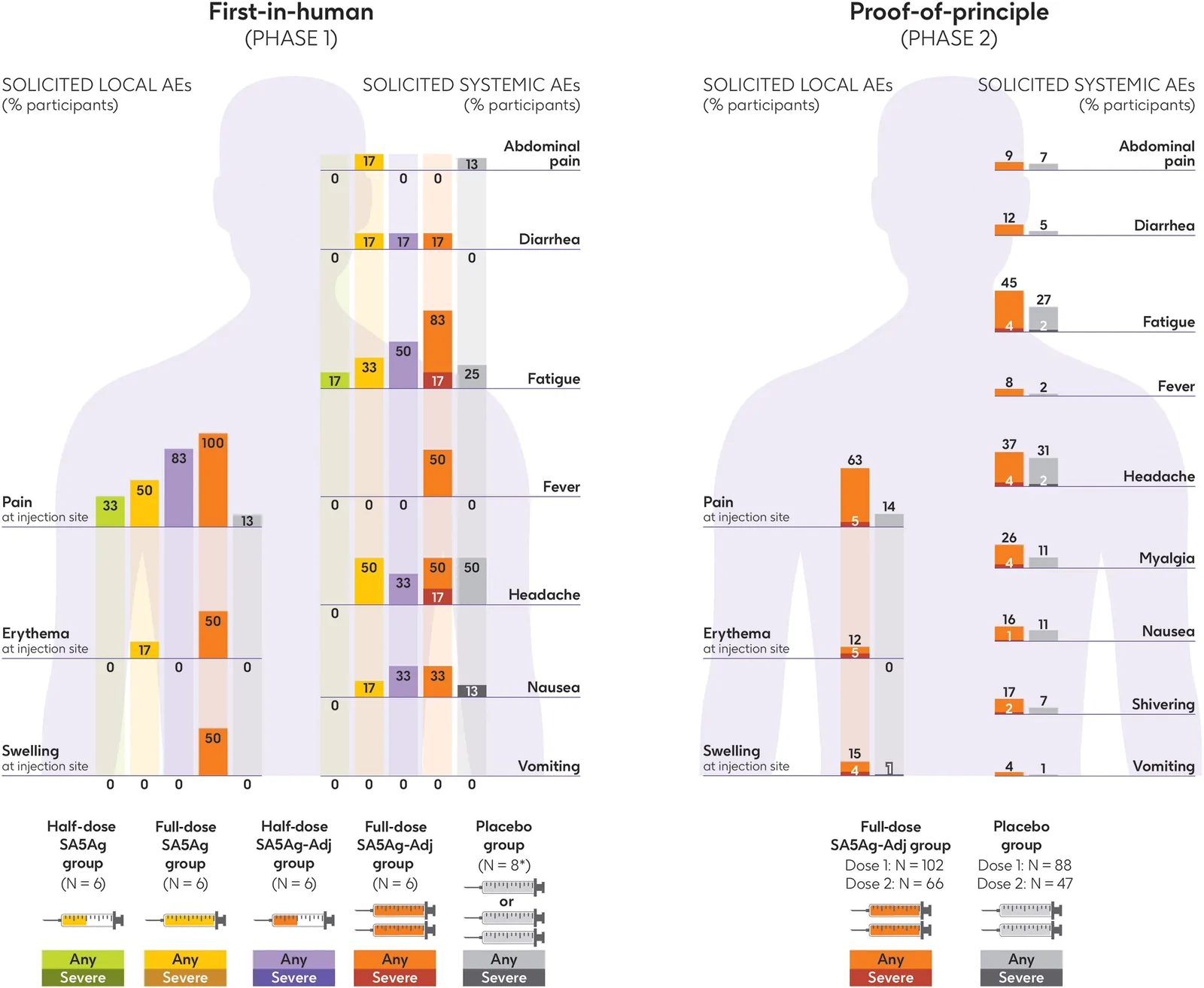
Descriptive analysis of solicited local and systemic AEs reported during the 7-day follow-up period after any vaccine/placebo dose in each study part. For SA5Ag and half-dose SA5Ag-Adj groups in phase 1: AEs after single dose. For all other groups: AEs after any of the 2 doses administered. *Pooled analysis of 6 participants who received a single dose (groups 1–3) and 2 participants who received 2 doses (group 4). “Severe” defined as preventing normal activity, apart from erythema and swelling (severe: diameter >100 mm), vomiting/diarrhea (≥6 episodes in 24 hours or requiring intravenous hydration), and fever (severe: >40°C). Abbreviations: AE, adverse event; N, number of participants in group; SA5Ag, nonadjuvanted 5-antigen *Staphylococcus aureus* vaccine formulation; SA5Ag-Adj, SA5Ag vaccine formulation adjuvanted with AS01_E_.

**Table 1.** Summary of Participants Reporting Unsolicited Adverse Events After Any Vaccine/Placebo Dose in Each Study Phase

Category	Follow-up Period After Last Dose	Dose-Escalation/First-in-Human Study (Phase 1), n (%)					Proof-of-Principle Study (Phase 2), n (%)	
		Half-Dose SA5Ag (Group 1; N = 6)^a^	Full-Dose SA5Ag (Group 2; N = 6)^a^	Half-Dose SA5Ag-Adj (Group 3; N = 6)^a^	Full-Dose SA5Ag-Adj (Group 4; N = 6)	Placebo (Groups 1–4; N = 8)^b^	SA5Ag-Adj Group (N = 103)	Placebo Group (N = 89)
Any unsolicited AE	30 days	1 (16.7)	3 (50.0)	1 (16.7)	2 (33.3)	0	40 (38.8)	27 (30.3)
Related	…	1 (16.7)	2 (33.3)	0	2 (33.3)	0	22 (21.4)	6 (6.7)
Severe	…	0	0	0	0	0	1 (1.0)	2 (2.2)
Related, severe	…	0	0	0	0	0	1 (1.0)	1 (1.1)
Medically attended unsolicited AEs	30 days	0	1 (16.7)	1 (16.7)	0	0	11 (10.7)	11 (12.4)
Serious AEs	12 months	0	0	0	0	0	5 (4.9)	5 (5.6)
Related	…	0	0	0	0	0	0	0
Potential immune-mediated disease	12 months	0	0	0	0	0	0	0
Deaths	12 months	0	0	0	0	0	0	0

Abbreviations: AE, adverse event; N, number of participants in group; n, number of participants in category; SA5Ag, nonadjuvanted 5-antigen *Staphylococcus aureus* vaccine formulation; SA5Ag-Adj, SA5Ag vaccine formulation adjuvanted with AS01_E_.

^a^AEs after single dose for groups 1–3; for all other groups, AEs after any of the 2 vaccine/placebo doses administered.

^b^Pooled analysis of 6 participants who received single dose (groups 1–3) and 2 participants who received 2 doses (group 4).

Unsolicited AEs in the 30-day post-vaccination period were reported by 40 (38.8%) participants in the SA5Ag-Adj group and 27 (30.3%) in the placebo group, with 22 (21.4%) and 6 (6.7%) participants, respectively, reporting an unsolicited AE considered related to vaccination (Table 1). Two unsolicited AEs reported as related to study intervention had severe intensity (headache in the SA5Ag-Adj group; fatigue in the placebo group). Medically attended unsolicited AEs were reported by 11 (10.7%) participants in the SA5Ag-Adj group and 11 (12.4%) in the placebo group, with pyrexia the only AE reported more than once (by 2 participants in the SA5Ag-Adj group).

During the entire study period, 5 participants in each group reported SAEs (7 SAEs in the SA5Ag-Adj group; 6 in the placebo group). None were considered related to vaccination. There were no reports of pIMD or deaths. Baseline geometric mean IL-2 and IL-17A levels (± standard deviation) were similar between the group of participants who experienced SAEs (0.21 ± 0.82 and 0.18 ± 0.44 pg/mL, respectively) and those who did not experience SAEs (0.22 ± 0.61 and 0.21 ± 0.48 pg/mL, respectively).

Hematology and biochemistry results did not indicate any safety signal associated with the investigational vaccine formulations tested in either study phase (data not shown).

### Efficacy: Secondary and Tertiary Objectives

The outcome of the interim analysis on 13 cases of recurrent SA-SSTI events reported from 14 days after dose 2 met the predefined futility criterion. The VE estimate was −74.8% (73% repeated CI, −680.6 to 36.6; Table 2), and the between-group difference in time to first SSTI recurrence (from 14 days after dose 2) was not statistically significant (*P* = .8042; Figure 3). New enrollments and additional dose administrations for enrolled participants were therefore stopped; for the final analysis, data collected by end-of-study were analyzed descriptively. The final analysis showed VE of −38.1% (95% CI, −245.8 to 40.9; mFAS) and −10.1% (95% CI, −203.4 to 57.7; per-protocol set [PPS]) in the prevention of recurrent SA-SSTI from 14 days after the second SA5Ag-Adj dose (Table 2). For SA-SSTI recurrences that occurred from 14 days after the first dose, VE estimates were −75.5% (95% CI, −295.4 to 17.5; FAS) and 2.3% (95% CI, −139.4 to 58.7; PPS; Table 2).

**Figure 3.**
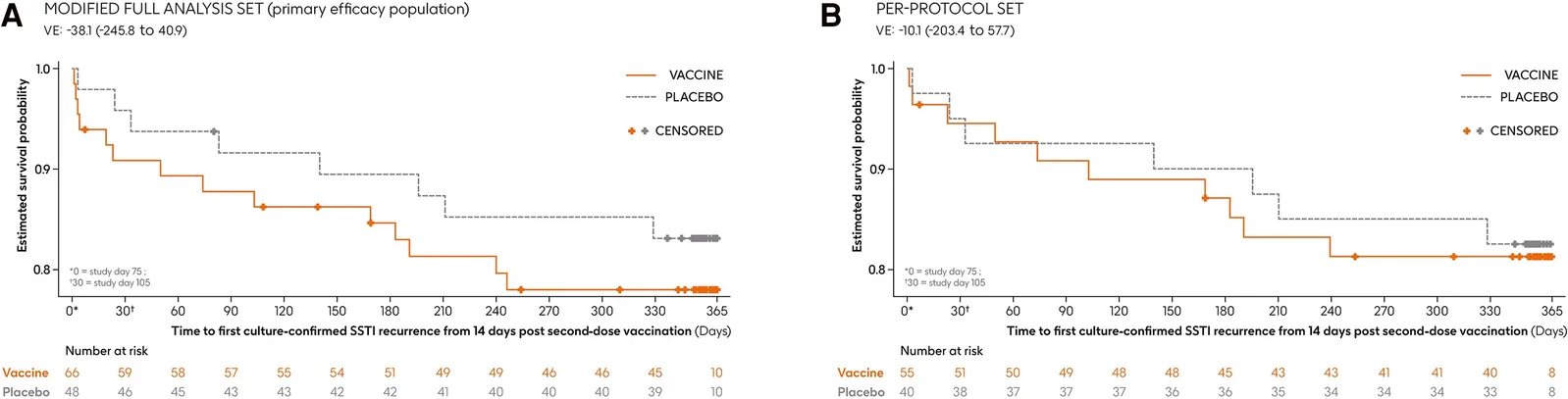
Kaplan–Meier curve of time to first culture-confirmed SA-SSTI recurrence from 14 days after the second SA5Ag-Adj dose, according to analysis population. Sample culture was conducted according to the standard of care at each participating institution's laboratory, which had Clinical Laboratory Improvement Amendments or equivalent accreditation.Abbreviation: SA5Ag-Adj, 5-antigen *Staphylococcus aureus* vaccine with AS01_E_ adjuvant; SA-SSTI, *Staphylococcus aureus* skin and soft tissue infection; SSTI, skin and soft tissue infection; VE, vaccine efficacy (final analysis ≥14 days after dose 2).

**Table 2.** Vaccine Efficacy of 5-Antigen *Staphylococcus aureus* Vaccine With AS01_E_ Adjuvant Against Recurrence of *Staphylococcus aureus* Skin and Soft Tissue Infections

Analysis Group	n	Person-Time, years	Incidence Rate Per 100 Person-Years (95% CI)	Hazard Ratio, %	Vaccine Efficacy, % (95% CI^a^)
Interim analysis ≥14 days after dose 2 (mFAS)					
Vaccine (N = 38)	10	10.6	94.7 (51.0 to 176.0)	174.8	−74.8 (−680.6 to 36.6)
Placebo (N = 21)	3	5.1	59.3 (19.1 to 183.9)	…	…
Final analysis ≥14 days after dose 2 (mFAS)					
Vaccine (N = 66)	14	50.9	27.5 (16.3 to 46.4)	138.1	−38.1 (−245.8 to 40.9)
Placebo (N = 48)	8	41.2	19.4 (9.7 to 38.9)	…	…
Final analysis ≥14 days after dose 2 (PPS)					
Vaccine (N = 55)	10	44.6	22.4 (12.1 to 41.7)	110.1	−10.1 (−203.4 to 57.7)
Placebo (N = 40)	7	34.9	20.1 (9.6 to 42.1)	…	…
Final analysis ≥14 days after dose 1 (FAS)					
Vaccine (N = 103)	18	90.4	19.9 (12.5 to 31.6)	175.5	−75.5 (−295.4 to 17.5)
Placebo (N = 89)	10	91.6	10.9 (5.9 to 20.3)	…	…
Final analysis ≥14 days after dose 1 (PPS)					
Vaccine (N = 55)	12	51.2	23.5 (13.3 to 41.3)	97.7	2.3 (−139.4 to 58.7)
Placebo (N = 40)	9	38.7	23.2 (12.1 to 44.7)	…	…

Abbreviations: CI, confidence interval; FAS, full analysis set; mFAS, modified full analysis set; N, number of participants; n, number of participants with at least 1 culture-confirmed recurrence of skin and soft tissue infection; PPS, per protocol set.

^a^CI for interim analysis: 92.5% lower limit, 80.5% upper limit. Repeated CIs for adjusting width of CIs to increase the joint coverage probability under any sequential monitoring scheme; lower repeated confidence level calculated from cumulative alpha spending, and upper repeated confidence level calculated from cumulative beta spending (nonbinding beta boundary). The 95% CI for the final analysis is based on a descriptive analysis (no alpha and beta control).

In both groups, most participants (13 of 15 [86.7%] in the SA5Ag-Adj group; 6 of 8 [75.0%] in the placebo group) had just 1 or 2 recurrent SA-SSTIs from 14 days after dose 2 to the end of 12-month follow-up, and an analysis of individual case report forms showed that almost 50% were reported within 3–4 months of the index SA-SSTI case. During the study, 1 participant (with medical history of SA-SSTI and drug abuse [substance use disorder]) in the SA5Ag-Adj group reported 7 recurrences, and 1 participant (with medical history of SA-SSTI and prediabetes) in the placebo group reported 5 recurrences; in both cases, recurrences were caused by methicillin-susceptible *S*. *aureus* (MSSA). Most of the recurrent SA-SSTIs were abscesses, followed by cellulitis, infected wounds, infected eczema, and infected ulcers.

An analysis that adjusted for history of SSTI and index methicillin-resistant *S*. *aureus* (MRSA) SSTI showed numerically more SSTI recurrences in the SA5Ag-Adj group than following placebo (Supplementary Table 2). Overall, 18 of 23 (78.3%) index SSTIs (11 in the vaccine group, 7 in the placebo group) were caused by MSSA and 5 (4 and 1, respectively) were caused by MRSA.

Baseline IL-2 concentrations for 23 participants with culture-confirmed SA-SSTI recurrences and 145 with no reported recurrence were (geometric mean ± standard deviation) 0.13 ± 0.46 and 0.24 ± 0.63 pg/mL, respectively. For IL-17A, these values were 0.12 ± 0.33 (25 participants) and 0.23 ± 0.49 pg/mL (133 participants), respectively.

### Immunogenicity: Tertiary Objective

Total immunoglobulin G (IgG) titers before vaccination were similar between groups and remained at prevaccination levels in the placebo group (Figure 4). In the SA5Ag-Adj group, IgG titers for CP5 and CP8 peaked 2 weeks after dose 1 (Figure 4), with mean geometric increases (MGIs) from baseline of 33.8 (95% CI, 23.2 to 49.2; mFAS) and 23.0 (95% CI, 14.2 to 37.3), respectively. For ClfA, Hla, and SpA, IgG titers peaked 2 weeks after dose 2 (ClfA: MGI, 85.4 and 95% CI, 62.5 to 116.6; Hla: MGI, 9.5 and 95% CI, 7.3 to 12.3; and SpA: MGI, 33.1 and 95% CI, 20.1 to 54.5; Figure 4). Antibody titers decreased during the 12-month follow-up but remained higher than baseline (MGI at month 14 from baseline ranged from 4.6 [95% CI, 2.9 to 7.3] for Hla to 24.9 [95% CI, 14.9 to 41.6] for anti-ClfA). Overall, humoral immune responses during the study were similar between participants with SA-SSTIs and those without recurrences. Functional antibody responses against Hla, CP5, and CP8 were above the assays' lower limit of quantification (LLOQ), while functional antibody titers against ClfA and SpA were below the LLOQ at most or all time points (maximum percentage of participants with functional titers ≥LLOQ, 2 weeks post-dose 1, was 39.6% for anti-ClfA, 9.1% for anti-SpA). No substantial differences were observed over time in the IgG avidity index against each antigen following SA5Ag-Adj exposure. Among the subset of 24 participants in the CMI analysis (11 in the SA5Ag-Adj group, 13 in the placebo group), vaccine-induced antigen-specific CD4+ T cells peaked 14 days after dose 1 in most vaccinated participants, with little further increase after dose 2. Hla induced a higher frequency of CD4+ T-cells than ClfA or SpA.

**Figure 4.**
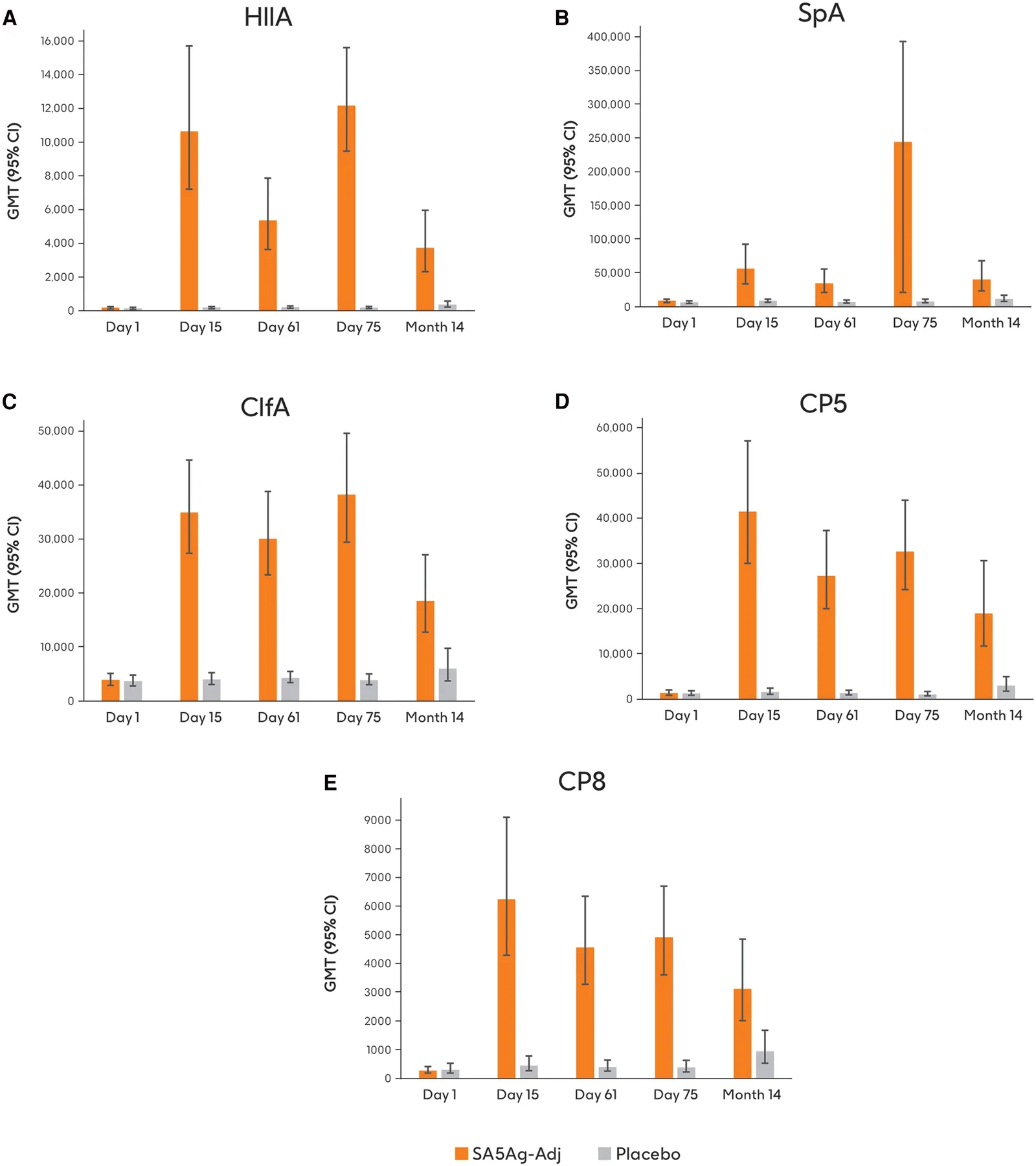
Immunoglobulin G geometric mean titers against each SA5Ag-Adj antigen. Abbreviations: CI, confidence interval; ClfA, clumping factor A; CP, capsular polysaccharide; GMT, geometric mean titer; Hla, alpha-hemolysin; SA5Ag-Adj, 5-antigen *Staphylococcus aureus* vaccine with AS01_E_ adjuvant; SpA, staphylococcal protein A.

## DISCUSSION

*Staphylococcus aureus* has been a target of vaccine development since the start of the 20th century [18]. Several unadjuvanted vaccine candidates were successfully tested in animal protection models and elicited good immune responses in early-phase clinical trials but failed to show protective efficacy in various human populations [8–14]. Consequently, adjuvants capable of eliciting T-cell–mediated responses have been included in *S*. *aureus* candidate vaccine formulations [15, 16]. GSK's SA5Ag-Adj investigational vaccine contains 5 antigens, which target 4 of the main *S*. *aureus* virulence factors [16] and showed protection against *S*. *aureus*–mediated infections in animal models [19–23], plus the adjuvant AS01_E_ [17].

After demonstrating acceptable safety in phase 1, enrollment of participants began with a recent SA-SSTI in phase 2. This was terminated early because prespecified futility criteria for the secondary efficacy endpoint were met at the interim analysis of 59 participants and overall 13 recurrent SA-SSTI cases reported after dose 2. All efficacy analyses showed that 1 and 2 doses of SA5Ag-Adj were not efficacious in preventing recurrent SA-SSTI, despite inducing robust functional immune responses against 3 (CP5, CP8, and Hla) of the 5 antigens in the vaccine formulation, and CMI responses. Almost half of recurrent SA-SSTIs were observed in the first 3–4 months after the index SA-SSTI case, with most index cases (78%) due to MSSA. No safety concerns relating to *S*. *aureus* vaccine administration were identified, and no SA-SSTI exacerbation was observed following SA5Ag-Adj exposure.

The low number of reported recurrences and the imbalanced randomization ratio (3:1, SA5Ag-Adj:placebo) for the first 40 participants in phase 2 may have contributed to the observed negative VE point estimates with wide CIs crossing zero. Recent medical history of SA-SSTI was adopted as an inclusion criterion to generate VE evidence in 1 of the populations of interest but might also partly explain the trial outcome. As reported in the literature [24–26] and highlighted by high recurrent infection rates in individuals with SSTIs [3, 27], immune responses following natural exposure to *S*. *aureus* do not confer adequate protection against infection. Mouse model data show that *S*. *aureus* infection may imprint nonprotective antibodies and interfere with vaccine-mediated immune responses [25, 26, 28]. As with the “original antigenic sin” theory postulated for other pathogens [29], animal model data and repeated unsuccessful clinical efficacy trials suggest that, for *S*. *aureus*, the first encounter matters, possibly triggering a nonprotective immune imprint that orientates subsequent responses via diverse mechanisms, including those mediated by IL-10, suppressing protective T-cell responses [24, 30, 31]. Also, it is noteworthy that 2 of the 3 protein antigens (ClfA and SpA) did not elicit the expected functional immune responses, and the other 3 antigens appear to have recalled nonprotective memory responses. Finally, antigenic diversity among circulating *S. aureus* strains and the pathogen's ability to sense body defense signals and modulate its virulence [32] might have contributed to the immune responses observed and the vaccine's inability to confer protection against SSTI. Further characterization of humoral and cellular responses to *S*. *aureus*, vaccination of different populations (eg, infants versus older individuals; asymptomatic carriers versus infected individuals), and preclinical models that accurately mimic *S*. *aureus*–human interactions might help improve our understanding of how, when, and who to target with vaccination against this “Janus-faced” bacterium [31–33].

Despite no immunological correlate of protection against *S*. *aureus* infections, this study had sufficient statistical strength to generate an early VE assessment in participants at risk of recurrent SA-SSTIs. However, our study was limited by being halted at the interim analysis, precluding the planned statistically powered efficacy examinations.

In conclusion, among participants with a recent history of SA-SSTI, SA5Ag-Adj vaccine showed an acceptable safety and tolerability profile, induced robust functional responses against CP5, CP8, and Hla antigens, but did not show efficacy in preventing recurrent SA-SSTIs.

## Supplementary Material

ciag162_Supplementary_Data

## Supplementary Data

Supplementary materials are available at *Clinical Infectious Diseases* online. Consisting of data provided by the authors to benefit the reader, the posted materials are not copyedited and are the sole responsibility of the authors, so questions or comments should be addressed to the corresponding author.

## Notes

***Staph aureus* 208833 Study Group**. Katherine Garnham, Fiona Bisshop, Mark Bloch, Olga Voloshyna, Brajesh Gupta, Kirankumar Jadhav, Krina Patel, Penelope Montgomery, Renate Koops, Joanne Finlay, Michal Adamczyk, Shaunagh Emanuel, Rosie Mngqibisa, Loren Miller, Scott Striplin, David Dougherty, and Nirav Shah.

***Author Contributions***. All authors participated in study design, implementation, or analysis; interpretation of the study; and development of the manuscript. All authors had full access to the data and gave final approval before submission.

***Acknowledgments***. All members of the *Staph aureus* 208833 Study Group were involved in the planning, conduct, and/or reporting of the work described in this article. We are grateful to the study participants and to GSK team members for their contributions to the study, particularly Annaelisa Tasciotti, Gennaro Di Maro, Carlo Curina, and Arthur Alban (safety); Anjali Pandey and Naveen Karkada (statistics); Darren Kelly, Jakub Skrobanek, and Adil Rakki (programming); Giovanna Curreli, Sheetal Verlekar, Kinga Szymborska, and Luigi Petillo (clinical operations); Silvia Rossi Paccani (preclinical team); Jones Lazar, Palak Bandiwar, and Priyanka Deshpande (data management); Venanzio Vella (epidemiology); and Fabio Bagnoli and Roberto Adamo (vaccine development).

The authors would like to thank Enovalife Medical Communication Service Center for editorial assistance, publication coordination, and writing support (Joanne Knowles, independent medical writer), on behalf of GSK.

***Trademark.*** AS01 is a trademark owned by or licensed to the GSK group of companies.

***Data sharing statement.*** Please refer to https://www.gsk-studyregister.com/en/ to access GSK's data sharing policies and as applicable seek anonymized subject level data.

***Financial support.*** This work was supported by GSK, which was involved in all stages of research conduct, including analysis of the data. GSK also took charge of all costs associated with the development and publication of this article.
