# Supplementary material for: Safety, Efficacy, and Immunogenicity of a Multivalent Adjuvanted Staphylococcus aureus Vaccine in Adults With Recent Skin and Soft Tissue Infections: An Observer-Blind, Randomized, Placebo-Controlled, Multinational Phase 1/2 Trial
Source: Clin Infect Dis. 2026 Mar 9;83(3):e569–77. doi: 10.1093/cid/ciag162 (PMC13644877; doi:10.1093/cid/ciag162)
Supplement: Supplementary file 1 [file ciag162_supplementary_data.docx]

**SUPPLEMENTARY MATERIAL**

**Safety, Efficacy, and Immunogenicity of a Multivalent Adjuvanted *Staphylococcus aureus* Vaccine in Adults With Recent Skin And Soft Tissue Infections: An Observer-Blind, Randomized, Placebo-controlled, Multinational Phase 1/2 Trial**

Eugene Athan,^1^ Richard N. Greenberg,^2^ David A Baker,^3^ Ruchir Shah,^4^ Siddharth Dubhashi,^5^ Aysha Badat,^6^ Claire Thurlow,^7^ Joel Schlessinger,^8^ Adam Wronski,^9^ Marcin Zakrzewski,^10^ David Turner,^11^ Ilaria Bindi,^12^ Venere Basile,^12^ Bruno Galletti,^12^ Fabiana Spensieri,^12^ Michela Brazzoli,^12^ Giulia Zigon,^12^ Giulia Luna Cilio,^12^ Giulia Ranzato,^12^ Maria Lattanzi,^12^ and Michele Pellegrini^12^; for the *Staph aureus* 208833 Study Group

^1^Public Health Unit, Barwon Health, Deakin University, Geelong, Australia; ^2^University of Kentucky, Lexington, KY, USA; ^3^East Sydney Doctors, Darlinghurst, NSW, Australia; ^4^Sanjivani Super Specialty Hospital, Ahmedabad, India; ^5^All India Institute of Medical Science, Nagpur, India; ^6^Wits Clinical Research, Soweto, Johannesburg, South Africa; ^7^Tasman, Southern Clinical Trials, Nelson, New Zealand; ^8^Advanced Skin Research Center, Omaha, NE, USA; ^9^Specjalistyczny Osrodek Deramatologi, Bialystok, Poland; ^10^Nzoz Holsamed, Oddzial Libero, Katowice, Poland; ^11^Cripps Health Centre, University of Nottingham, United Kingdom; and ^12^GSK, Siena, Italy.

**Correspondence**: M. Pellegrini, GSK, Via Fiorentina 1, 53100 Siena, Italy (michele.x.pellegrini@gsk.com).

**Table of contents**

[Key inclusion/exclusion criteria	3](#_Toc218675510)

[Vaccine formulations	4](#_Toc218675511)

[Assessment of safety and reactogenicity endpoints	4](#_Toc218675512)

[Assessment of immunogenicity endpoints	5](#_Toc218675513)

[Details of statistical analysis	6](#_Toc218675514)

[Figure 1. Disposition of study participants in proof-of-principle part of study (phase 2).	7](#_Toc218675515)

[Table 1. Demographic characteristics of participants in each part of the phase 1/2 study.	8](#_Toc218675516)

[Table 2. Number of culture-confirmed occurrences of skin and soft tissue infection (SSTI) after dose 2, adjusted for previous SSTI and methicillin-resistant *Staphylococcus aureus* type of index SSTI.	10](#_Toc218675517)

## Key inclusion/exclusion criteria

See study protocol for full listing of inclusion/exclusion criteria.

All participants had to satisfy all of the following inclusion criteria at study entry:

- Male or female participants aged (for first-in-human, phase 1) between 18 and 50 years or (for proof-of-principle, phase 2) between 18 and 64 years, inclusive, at the time of first vaccination, who, in the opinion of the investigator, can and will comply with the requirements of the protocol (e.g. completion of the diary cards, return for follow-up visits).
- Written or witnessed informed consent obtained from the participant prior to performance of any study-specific procedure.
- Female individuals of non-childbearing potential could be enrolled in the study. Female individuals of childbearing potential could be enrolled if the individual had practiced adequate contraception for 30 days prior to vaccination, a negative pregnancy test on the day of enrollment, and agreed to continue adequate contraception during the entire treatment period and for 2 months after completion of the vaccination series.

Additional inclusion criterion for participants to be enrolled in the first-in-human, phase 1:

- Healthy individuals, as established by medical history, clinical examination, and laboratory assessment.

Additional inclusion criteria for participants to be enrolled in the proof-of-principle, phase 2:

- Healthy individuals, as established by medical history and clinical examination before entering into the study, with an ongoing *Staphylococcus aureus* skin and soft tissue infection (SSTI), i.e., *S*. *aureus* was the most likely cause, as confirmed by an *S*. *aureus*-positive culture not earlier than 30 days prior to informed consent form signature. Before randomization, participants had to be treated until clinical resolution of the culture-confirmed SSTI caused by *S*. *aureus*.

Exclusion criteria for enrollment:

- Body mass index (BMI) >40 kg/m^2^.
- History of any reaction or hypersensitivity likely to be exacerbated by any component of the vaccine.
- Hypersensitivity to latex.
- Recurrent history of uncontrolled neurological disorders or seizures.
- History of any serious chronic or progressive disease according to the judgment of the investigator (e.g., neoplasm; insulin-dependent diabetes; cardiac, renal, or hepatic disease).
- History of potential immune-mediated disease.
- Clinical conditions that, in the investigator’s opinion, represent a contraindication to intramuscular vaccination and blood draws.
- Any clinical condition that, in the investigator’s opinion, might pose an additional risk to an individual due to their participation in the study.

Exclusion criteria considered at time of vaccination in proof-of-principle, phase 2:

- Any clinically significant hematological (hemoglobin level; white blood cell, lymphocyte, neutrophil, eosinophil, platelet count, or red blood cell count) and/or biochemical (alanine aminotransferase, aspartate aminotransferase, creatinine) laboratory abnormality at the time of vaccination.
- Microbiological test results of drainage suggesting that the SSTI etiology could be due to an infection other than with *S*. *aureus* (i.e., culture shows presence of another pathogen that, in the investigator’s opinion, could have caused the SSTI).

## Vaccine formulations

A full SA5Ag vaccine dose (0.5 mL injectable volume) contained two bioconjugates (capsular polysaccharide type 5, 8 μg, conjugated to detoxified alpha-hemolysin, 60 μg; capsular polysaccharide type 8, 8 μg, conjugated to mutated clumping factor A, 60 μg) and mutated staphylococcal protein A (60 μg). The adjuvanted formulations contained AS01_E_, an Adjuvant System containing 3-*O*-desacyl-4′-monophosphoryl lipid A (MPL), QS-21, and liposome (25 μg MPL and 25 μg QS-21). Half-dose and full-dose formulations were derived by resuspending the lyophilized product with appropriate volumes of saline (unadjuvanted formulations) or AS01_E_ (adjuvanted formulations).

## Assessment of safety and reactogenicity endpoints

Primary reactogenicity and safety endpoints included percentages of group participants reporting solicited local (pain, erythema, swelling at the injection site) or systemic (headache, fatigue, nausea, vomiting, diarrhea, abdominal pain, myalgia, shivering, fever) adverse events (AEs), recorded in electronic diaries for 7 days after each dose. Solicited AEs were classified as having mild, moderate, or severe intensity, with severe defined as preventing normal everyday activities, apart from erythema and swelling for which severe was defined as diameter >100 mm and vomiting/diarrhea for which severe was defined as ≥6 episodes in 24 hours or requiring intravenous hydration. Fever was defined as body temperature ≥38.0°C and severe fever as >40°C. Unsolicited AEs reported up to 30 days after each dose, and serious AEs (SAEs), medically attended AEs, and potential immune-mediated disease (pIMD) in the 12 months following the last study dose, were reported by Medical Dictionary for Regulatory Activities system organ class and preferred term. The causal relationship of unsolicited AEs/SAEs to vaccination was assessed by study investigators. Additional primary endpoints for the first-in-human study part (phase 1) and for the first 40 participants in the proof-of-principle part (phase 2) were percentages of participants with hematological and biochemical laboratory abnormalities and changes from baseline values (blood sample taken before dose administration) after vaccination.

## Assessment of immunogenicity endpoints

All immunogenicity assays listed in the table below were customized, to allow descriptive analyses of the tertiary immunogenicity endpoints. Total immunoglobulin G (IgG) concentrations and functional antibody titers were assessed at study days 1, 15, 61, 75, and month 14; cell-mediated immunity was assessed at days 1, 15, and 75.

| **Antigen** | **Total IgG concentration** | **Functional immunogenicity** | **Cell-mediated immunity*** |
| --- | --- | --- | --- |
| **CP5** | Multiplex (5-plex) assay^†^ | CP5 opsonophagocytic assay | Not applicable |
| **CP8** | Multiplex (5-plex) assay^†^ | CP8 opsonophagocytic assay | Not applicable |
| **ClfA** | Multiplex (5-plex) assay^†^ | ClfA binding inhibition assay | CFC-ICS |
| **Hla** | Multiplex (5-plex) assay^†^ | Hla hemolysis inhibition assay | CFC-ICS |
| **SpA** | Multiplex (5-plex) assay^†^ | Antibody-mediated SpA binding assay | CFC-ICS |

* Frequency of Hla-, ClfA-, and SpA-specific CD4+/CD8+ T cells

^†^ Luminex technology

CFC-ICS, cell-flow cytometry combined with intracellular cell staining; Clf, clumping factor; CP, capsular polysaccharide; Hla, alpha-hemolysin; SpA, staphylococcal protein A.

**Interleukin 2 (IL-2), IL-17A testing:** performed using Simoa™ commercial digital enzyme-linked immunosorbent assay at pre-vaccination (day 1) assessment.

## Details of statistical analysis

The phase 2 proof-of-principle part had a group sequential, event-driven design, allowing an interim analysis for efficacy/futility with 13 events of recurrent SA-SSTIs, provided 27 events were required for the final analysis, considering a one-sided alpha equal to 7.5% and power equal to 80% and an estimated vaccine efficacy (VE) of 61% in the SA5Ag-Adj group 2 weeks after the second vaccine dose with the lower limit >0% for the 85% confidence interval. For the calculation of number of participants, an SA-SSTI recurrence incidence rate of 7% in the placebo group was considered.

Analysis of the key secondary endpoint for efficacy was based on the modified full analysis set (mFAS; participants who received two doses and provided efficacy data), with two planned sequential analyses following a group sequential design using the O’Brien-Fleming spending function (with 48% and 100% of the total planned events), with an overall cumulative one-sided alpha of 7.5% and cumulative beta of 20%. Log-rank score statistics and boundary values in the score scale were used to test for early stopping at the interim analysis. This interim analysis for efficacy/futility was performed when 13 cases of recurrent SA-SSTI were reported in the 14-day period after the second SA5Ag-Adj dose. A test statistic value higher than the alpha boundary value (3.711 in the score scale) would show the efficacy criteria had been met, a value between the alpha and beta boundaries would suggest the study should continue, and a value lower than the beta boundary value (0.350 in the score scale) would suggest the futility criteria had been met at the interim analysis (non-binding criteria). The between-group hazard ratio and its repeated CIs were calculated using observed statistics and boundary values, with VE derived as 100 × (1 minus hazard ratio). A one-sided P-value for the log-rank test was calculated to check for statistical significance. The one-sided nominal type I error was 1.61% at the interim analysis and 5.89% at the final analysis, with no early stop at the interim.

The log-rank test statistic (-1.462 in the score scale) was calculated at the interim analysis using 1.5% alpha and 8.1% beta. The alpha and beta spent at interim were dependent on information accrued from up to 13 recurrent SA-SSTI events.

Similarly, the VE objective was to be assessed for all events occurring in the full analysis set (participants who received at least one dose of SA5Ag-Adj or placebo and for whom efficacy data were available) according to the group sequential test but, since statistical significance for the key secondary endpoint was not demonstrated, no hypothesis testing was performed for this co-secondary endpoint and a descriptive analysis was provided. Analyses of the per-protocol set, i.e., excluding those participants with major protocol violations, were also conducted.

For the proof-of-principle part of the study (phase 2), a descriptive analysis of humoral immunogenicity following SA5Ag-Adj or placebo was conducted on the mFAS (participants who received at least one dose and provided immunogenicity data). Antibody IgG avidity (multiplex immunoassay) and functional antibody concentrations were determined in participants who received two doses. Serum levels of IL-1 and IL-17A were tested for all participants at baseline. Cell-mediated immunity was tested descriptively in a subset of 24 participants who consented to an additional blood draw for peripheral blood mononuclear cell isolation.

## Figure 1. Disposition of study participants in proof-of-principle part of study (phase 2).

AE, adverse event; N, number of participants; n, number of participants in category; SAE, serious adverse event; SA5Ag-Adj, five-antigen *Staphylococcus aureus* vaccine with AS01_E_ adjuvant.

Alt text: Flow chart showing the distribution of participants in the proof-of-principle part of study, from enrollment to study completion.

## Table 1. Demographic Characteristics of Participants in Each Part of the Phase 1/2 Study.

| **Characteristic** | **Dose-Escalation/First-in-Human Study Part (Phase 1)** | | | | | **Proof-of-Principle Study Part (Phase 2)** | |
| --- | --- | --- | --- | --- | --- | --- | --- |
|  | **Half-Dose SA5Ag (Group 1; N=6)** | **Full-Dose SA5Ag (Group 2; N=6)** | **Half-Dose SA5Ag-Adj (Group 3; N=6)** | **Full-Dose SA5Ag-Adj (Group 4; N=6)** | **Placebo (Groups 1–4; N=8)** | **SA5Ag-Adj Group (N=103)** | **Placebo Group (N=89)** |
| Age (years), mean (SD) | 26.3 (3.1) | 31.0 (11.5) | 34.5 (7.7) | 30.8 (6.5) | 32.5 (9.1) | 38.2 (13.5) | 38.7 (13.4) |
| Sex, n (%) |  |  |  |  |  |  |  |
| Male | 3 (50) | 1 (16.7) | 1 (16.7) | 4 (66.7) | 2 (25.0) | 59 (57.3) | 55 (61.8) |
| Female | 3 (50) | 5 (83.3) | 5 (83.3) | 2 (33.3) | 6 (75.0) | 44 (42.7) | 34 (38.2) |
| Country, n (%) |  |  |  |  |  |  |  |
| United States | 6 (100) | 6 (100) | 6 (100) | 6 (100) | 8 (100) | 10 (9.7) | 9 (10.1) |
| India | - | - | - | - | - | 31 (30.1) | 31 (34.8) |
| Australia | - | - | - | - | - | 26 (25.2) | 15 (16.9) |
| New Zealand | - | - | - | - | - | 19 (18.4) | 11 (12.4) |
| Poland | - | - | - | - | - | 6 (5.8) | 8 (9.0) |
| South Africa | - | - | - | - | - | 9 (8.7) | 12 (13.5) |
| United Kingdom | - | - | - | - | - | 2 (1.9) | 3 (3.4) |
| Race, n (%) |  |  |  |  |  |  |  |
| White | 6 (100) | 6 (100) | 4 (66.7) | 5 (83.3) | 7 (87.5) | 53 (51.5) | 37 (41.6) |
| Asian | 0 | 0 | 0 | 1 (16.7) | 0 | 34 (33.0) | 33 (37.1) |
| Black or African American | 0 | 0 | 2 (33.3) | 0 | 1 (12.5) | 11 (10.7) | 12 (13.5) |
| Native Hawaiian or other Pacific islander | 0 | 0 | 0 | 0 | 0 | 4 (3.9) | 0 |
| Other | 0 | 0 | 0 | 0 | 0 | 1 (1.0) | 7 (7.9) |

SA5Ag, non-adjuvanted five-antigen *Staphylococcus aureus* vaccine formulation; SA5Ag-Adj, SA5Ag vaccine formulation adjuvanted with AS01_E_; N, number of participants in group; n, number of participants in category; SD, standard deviation.

## Table 2. Number of Culture-Confirmed Occurrences of Skin and Soft Tissue Infection (SSTI) After Dose 2, Adjusted for Previous SSTI and Methicillin-Resistant *Staphylococcus aureus* Type of Index SSTI.

| **Group** | **n** | **Person-Time (Years)** | **Incidence Rate per 100 Person-Years** | **Incidence Rate Ratio per 100 Person-Years (95% CI)** |
| --- | --- | --- | --- | --- |
| SA5Ag-Adj (N=67) | 15 | 63.0 | 3.5 | 160.8 (69.3–372.9) |
| Placebo (N=48) | 8 | 47.1 | 2.2 |  |

CI, confidence interval; N, number of participants who received two doses; n, number of participants with at least one culture-confirmed recurrence of SSTI; SA5Ag-Adj, five-antigen *Staphylococcus* *aureus* vaccine with AS01_E_ adjuvant.
